# Editorial: Feedlot cattle nutrition and metabolism

**DOI:** 10.3389/fvets.2023.1239768

**Published:** 2023-06-27

**Authors:** Danilo Domingues Millen

**Affiliations:** Department of Animal Science, São Paulo State University, Jaboticabal, São Paulo, Brazil

**Keywords:** acidosis, cattle, feedlot, heat stress, additives

Like other ruminants, cattle evolved as herbivores by consuming forages (grasses and legumes), characterized by the high cell wall content. As a result, the rumen microbiota was shaped over the centuries by fermenting structural carbohydrates, turning the rumen into an environment rich in fiber-utilizing microorganisms, including bacteria, protozoa, and fungi. However, high levels of productivity cannot be achieved when cattle are consuming forage-based diets, and to increase beef production, cattle are often fed and finished in feedlot operations.

Feedlot cattle are often fed highly fermentable diets to achieve rapid rates of gain and improve feed efficiency. However, this type of diet causes excessive rates of acid production in the rumen, which can overwhelm the ability of cattle to regulate ruminal pH, and consequently may increase the risk of ruminal acidosis. Based on this fact, the objective of this Research Topic was to collect suitable papers to improve our knowledge and understanding of rumen function and metabolism in order to mitigate acidosis and improve the performance of feedlot cattle.

Most articles on this Research Topic addressed the effects of some type of feed additive, or a combination of them, to manipulate ruminal fermentation and maximize either the short-chain fatty acids production in the rumen or the animal performance. Combinations of feed additives to prevent ruminal disorders and increase productivity included: monensin and narasin (Baggio et al.), monensin and essential oils (Silva et al.), monensin and virginiamycin (Rigueiro et al.). Sodium monensin is the most used feed additive in feedlot diets around the world, however, due to its negative effect on dry matter intake, the association with other feed additives may lead to a synergistic effect to improve cattle health and performance.

Moreover, the search for an alternative to replacing sodium monensin in feedlot diets, since its use as a growth promoter was banned in some countries, was also the objective of some of the authors on this Research Topic. Polyclonal antibodies failed on controlling ruminal acidosis when compared to monensin (Pacheco, Souza, et al.); however, virginiamycin was as effective as monensin to prevent rumen pH decline when cattle were adapted for 14 days (Squizatti et al.). Furthermore, a blend of essential oils and 25-hydroxyvitamin D3 seems to be an alternative to replace monensin in feedlot diets offered to Holstein steers (Latack et al.).

Interestingly, two papers addressed the effect of different molecules on reducing heat stress in feedlot cattle, which is literally a hot topic in production systems that are not even close to the natural habitat cattle are used to. Carvalho et al. reported that the use of NutraGen supplement induced significant changes in the metabolism of the steers, whereas Pacheco, Oliveira Gusmão, et al. concluded that lysolecithin enhances feedlot performance and has the potential to increase diet intake during very hot days.

In summary, this Research Topic contributed to improving the current knowledge of feedlot cattle nutrition and metabolism by providing an enormous amount of new relevant data that certainly are useful worldwide.

## Author contributions

DM idealized the Research Topic and wrote the manuscript.

